# A historical review and bibliometric analysis of research on fracture nonunion in the last three decades

**DOI:** 10.1007/s00264-021-05020-6

**Published:** 2021-04-18

**Authors:** Peter V. Giannoudis, George D. Chloros, Yuh-Shan Ho

**Affiliations:** 1 Academic Department of Trauma and Orthopaedics, School of Medicine, University of Leeds, Leeds, UK; 2 NIHR Leeds Biomedical Research Center, Chapel Allerton Hospital, Leeds, UK; 3 Trend Research Centre, Asia University, No. 500, Lioufeng Road, Wufeng, Taichung, 41354 Taiwan

**Keywords:** Nonunion, Fracture, Scientometrics, SCI-EXPANDED, Front page

## Abstract

**Background:**

Nonunion continues to be the most frequent and challenging complication to treat following fracture fixation. Herein, we carried out a bibliometric analysis aiming to identify the key researchers, centres and research trends developed during the past 30 years in this important clinical condition.

**Methods:**

The Science Citation Index Expanded database and the Web of Science Core Collection were interrogated for manuscripts published between 1990 and 2019 in the topic domain, utilising title, abstract, author keywords and KeyWords Plus. Overall, such citation indicators were used as TC_year_, C_year_ and CPP_year_ to help analyse the identified manuscripts.

**Results:**

Over the prespecified period, there was a steady increase in the number of articles published in fracture nonunion. In total, 12 languages were the primary languages in the documents, with English being the most prevalent. The CPP sharply increased to reach a plateau in three full years and up to a peak in ten full years. A total of 8976 nonunion-related articles in Science Citation Index Expanded (SCI-EXPANDED) were published in 790 journals. The 8976 articles were published by 26,079 authors among 101 different countries. There is a slightly fluctuating steady increase of articles from 116 in 1991 to 201 in 2003, and thereafter, the number of articles sharply increased to reach a plateau in 2015. Seven possible main research foci in nonunion-related research were identified including: epidemiology, classification, aetiology, diagnosis/prediction, treatment modalities, functional outcomes and health economics.

**Conclusions:**

This bibliometric analysis revealed information on citation number, publication outputs, categories, journals, institutions, countries, research highlights and tendencies. The current research activity on fracture nonunion identified key opinion leaders and leading research institutions focusing on this important clinical condition. It is hoped that the informed included will aid to guide research work in the foreseeable future.

## Introduction

Failure of fracture healing with resultant nonunion remains a very challenging but potentially devastating complication following fracture surgery and has an estimated incidence of about 5–10% [1]. Despite intense research efforts, this problem continues to take a significant toll on society, including both direct and indirect costs and major disability [2]. For example, nonunion of tibial shaft fractures may have an impact to the physical health worse than other common chronic condition such as congestive heart failure, back pain, myocardial infarction, diabetes and others [2].

Bibliometric citation analysis is an established evidence-based method of mapping the literature and identifying prominent researchers and research units that have developed a particular interest in a specific topic or condition and has been commonly used in orthopaedic surgery [3]. By analysing research output per country, institutions, journals and researchers, bibliometric analysis evaluates and improves the understanding of research trends evolution over time [3]. Although the orthopaedic community has remained active in addressing this challenge, it appears that fracture nonunion research is carried out within certain specialised groups and societies. The aim of this study therefore is to carry out a quantitative description of the literature on nonunion manuscripts published until now and to gather information on institutions, journals, researchers, countries, Web of Science categories and research directions. Moreover, we wished to outline an up-to-date, clear framework of the current research and provide clues to guide the future direction of the research in this yet unsolved clinical condition.

## Methods

Data were retrieved from the Science Citation Index Expanded (SCI-EXPANDED) in the Web of Science Core Collection by Clarivate Analytics and last updated on 10th February 2021. Although, by design, the Science Citation Index Expanded (SCI-EXPANDED) is useful for literature search [4], the Web of Science database may be queried and serve as basis to establish bibliometric searches. The following keywords were used to search in SCI-EXPANDED: ‘nonunion’, ‘nonunions’, ‘non union’ and ‘non unions’. The following topic search strings were used in Web of Science Core Collection: paper title, abstract and author keywords. Search power was enhanced using KeyWords Plus which provided additional search terms extracted from the titles of papers cited in each new article listed in Current Contents [5]. As the documents that can only be found by KeyWords Plus are irrelevant to the search topic [6], those were excluded. To eliminate inherent bias of using Web of Science Core Collection for bibliometric analysis and avoid introducing unrelated publications, the ‘front-page’ filter was used to cover only the documents in which search keywords are included in the title, abstract and author keywords [6]. By using advanced search with terms of TI (title), AB (abstract) and AK (author keywords), 10,064 documents including 8976 articles having the search keywords in their ‘front page’ from 1990 to 2019 were defined as nonunion publications. Subsequently, the records were introduced into spreadsheet software (Microsoft Excel 2016, Microsoft Corporation™), and additional coding was performed for analysis [6]. The journal impact factor (IF_2019_) for each journal was obtained from the Journal Citation Reports (JCR) in 2019. For accuracy and simplicity, affiliations of England, Scotland, Northern Ireland and Wales were reclassified as United Kingdom (UK) [6], whereas affiliations in Hong Kong prior to 1997 were included under the heading of China [6]. Affiliations from the former USSR were checked and reclassified as being from Ukraine and Russia, respectively, whereas those from Serbia and Montenegro were grouped as Serbia [6]. In the SCI-EXPANDED database, the corresponding author is labelled as reprint author, but in this study, we used the term corresponding author [6]. In a single-institution article, the institution is classified as the first and the corresponding author’s institution [6]. In multi-corresponding-author articles, only the last corresponding author, institution and country were assigned to the article [6]. Noteworthy, it has been shown previously that the corresponding author is most likely to appear first and then last in the byline [6].

The following three citation indicators were used to investigate the citations of the publications:

1. C_year_—the number of citations from the Web of Science Core Collection in a particular year. For example, C_2019_ refers to the number of citations in 2019 [7].
2. TC_year_—the total number of citations from the Web of Science Core Collection since publication year to the end of the most recent year, it is 2019 in this study [8]. This citation indicator has been commonly applied in bibliometric research in the last decade [9].
3. CPP_year_—citations per publication (CPP_2019_ = TC_2019_/TP); TP, total number of articles [7].

The following six publication indicators were applied to evaluate publication performance of countries and institutions [10]:

1. TP—total number of articles
2. IP—number of single-country or single-institution authored articles
3. CP—number of internationally or inter-institutionally collaborative articles
4. FP—number of first-author articles
5. RP—number of corresponding-author articles
6. SP—number of single-author articles

## Results and discussion

### Document type and language of publication

The relationship among document types and their citations per publication, CPP_year_, and the APP has recently been proposed [11]. Among the 14 document types indexed by the Web of Science, a total of 10,064 nonunion-related publications were found (Table 1). The document type of ‘articles’ was by far the most prevalent, with a total of 8976 articles (89% of 10,064 documents) and an average of 4.7 APP with a maximum of 200. The document type of the ‘proceedings’ papers had the highest CPP_2019_ of 41, which can be attributed to the 33 highly cited proceedings papers (11% of 306 proceedings papers) with TC_2019_ of 100 or more [6]. Of note, in the Web of Science document classification, one document may be classified in several categories. For example, 306 documents are classified as document types for ‘proceedings papers’ and ‘articles’, so the sum of the percentages is higher than 100% [6]. Only the 8976 articles were further analysed. In total, 12 languages were the primary languages in the articles, with English, as the most prevalent (93% of the total of 8976 articles), followed by German (324 articles; 3.6% of 8976 articles) and French (178; 2.0%). Minority languages were Czech (36 articles), Turkish (25), Portuguese (6), Spanish (5), Serbian (4), Italian (3), Russian (3), Polish (2) and Hungarian (1). English language articles had higher CPP_2019_ of 23 than non-English with CPP_2019_ of 7.5.

**Table 1:** Nonunion citations and authors according to the document type

Document type	*TP*	%	TP*	*AU*	*APP*	*TC*_2019_	*CPP*_2019_
Article	8976	89	8976	41,914	4.7	194,140	22
Review	683	6.8	683	2902	4.2	19,319	28
Proceedings paper	306	3.0	306	1393	4.6	12,691	41
Letter	137	1.4	137	305	2.2	348	2.5
Meeting abstract	122	1.2	118	601	5.1	35	0.29
Editorial material	97	1.0	96	249	2.6	699	7.2
Note	27	0.27	27	69	2.6	310	11
Correction	15	0.15	15	59	3.9	8	0.53
Retracted publication	7	0.070	7	37	5.3	122	17
Book chapter	4	0.040	4	21	5.3	54	14
Reprint	3	0.030	3	13	4.3	48	16
News item	2	0.020	0	0	N/A	0	0
Discussion	1	0.010	1	1	1.0	1	1.0
Retraction	1	0.010	1	5	5.0	0	0

*TP*, total number of publications; TP*, total number of publications with author information in SCI-EXPANDED; *AU*, number of authors; *APP*, number of authors per publication; *TC*_2019_, the total number of citations from Web of Science Core Collection since publication year to the end of 2019; *CPP*_2019_, number of citations (*TC*_2019_) per publication (*TP*); N/A, not available

### Historical publication and citation trends

**Fig. 1:**
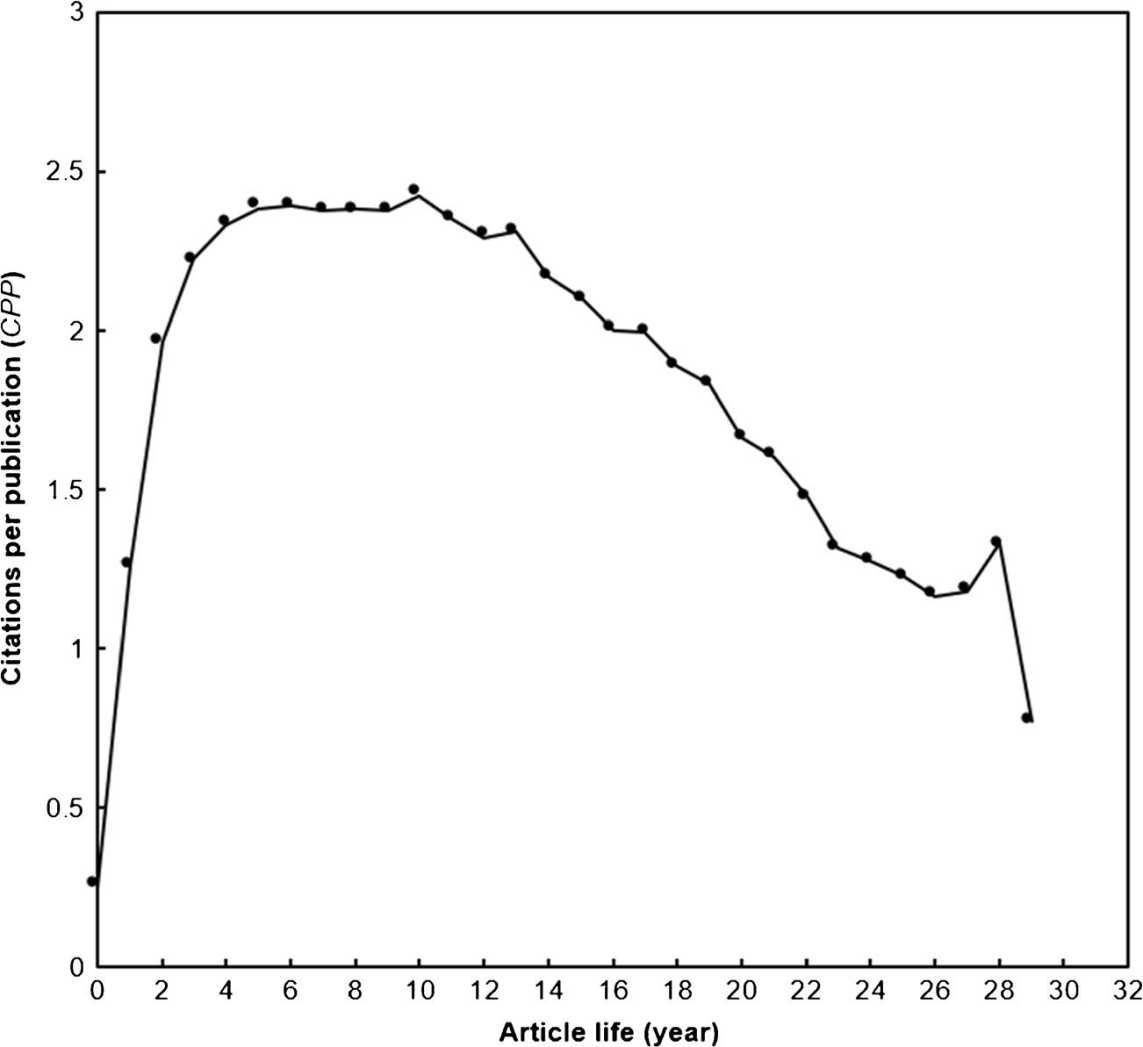
Citations per publication by article life

To understand publications in a specific research topic and their citation trends, a relationship between citations per publication (CPP_year_) and article life was proposed [12]. The article life with CPP for all the 8976 nonunion articles is displayed in Fig. 1. It takes CPPs about three full years to reach a plateau and up to a peak in ten full years. A comparison of the medical-related topics was also shown in Fig. 1. Articles related to nonunion had longer citation life than some medical topics; a peak appeared in four and five full year for breast reconstruction [6] and child sexual abuse [13] research, respectively.

**Fig. 2:**
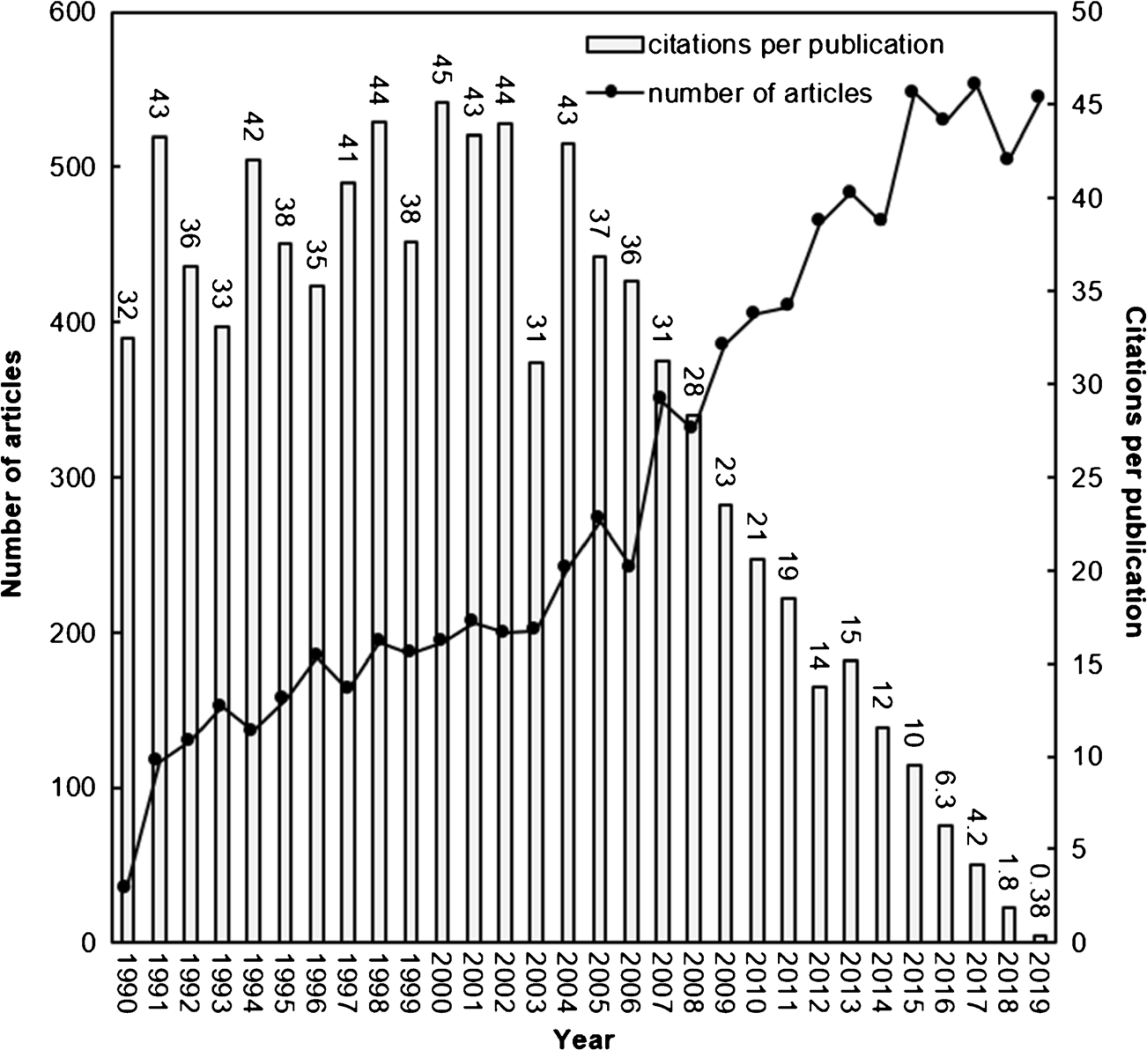
The number of nonunion articles and citations per publication by year

Both publications and citation trends in a research topic were proposed [6]. A relationship between the annual number of articles (TP) and their citations per publication (CPP_2019_ = TC_2019_/TP) throughout the years is shown in Fig. 2. The ‘jump’ between 1990 and 1991 is an artefact that arises from a policy change by the Web of Science with respect to abstract information [14]. The majority of articles have no abstract information in SCI-EXPANDED before 1991. There is a slightly fluctuating steady increase of articles from 116 in 1991 to 201 in 2003 and thereafter, the number of articles sharply increased to reach a plateau in 2015, fluctuating in the mid-550s. Based on Fig. 2, it takes CPPs about 14 years to reach a plateau. Articles related to nonunion had longer life than some medical-related topics, for example, breast reconstruction with 10 years[6] and child sexual abuse with 8 years [13]. Articles published in nonunion could be cited longer with average year of 14 than that of breast reconstruction and child sexual abuse with ten and eight years, respectively.

### Web of Science categories and journals

**Fig. 3:**
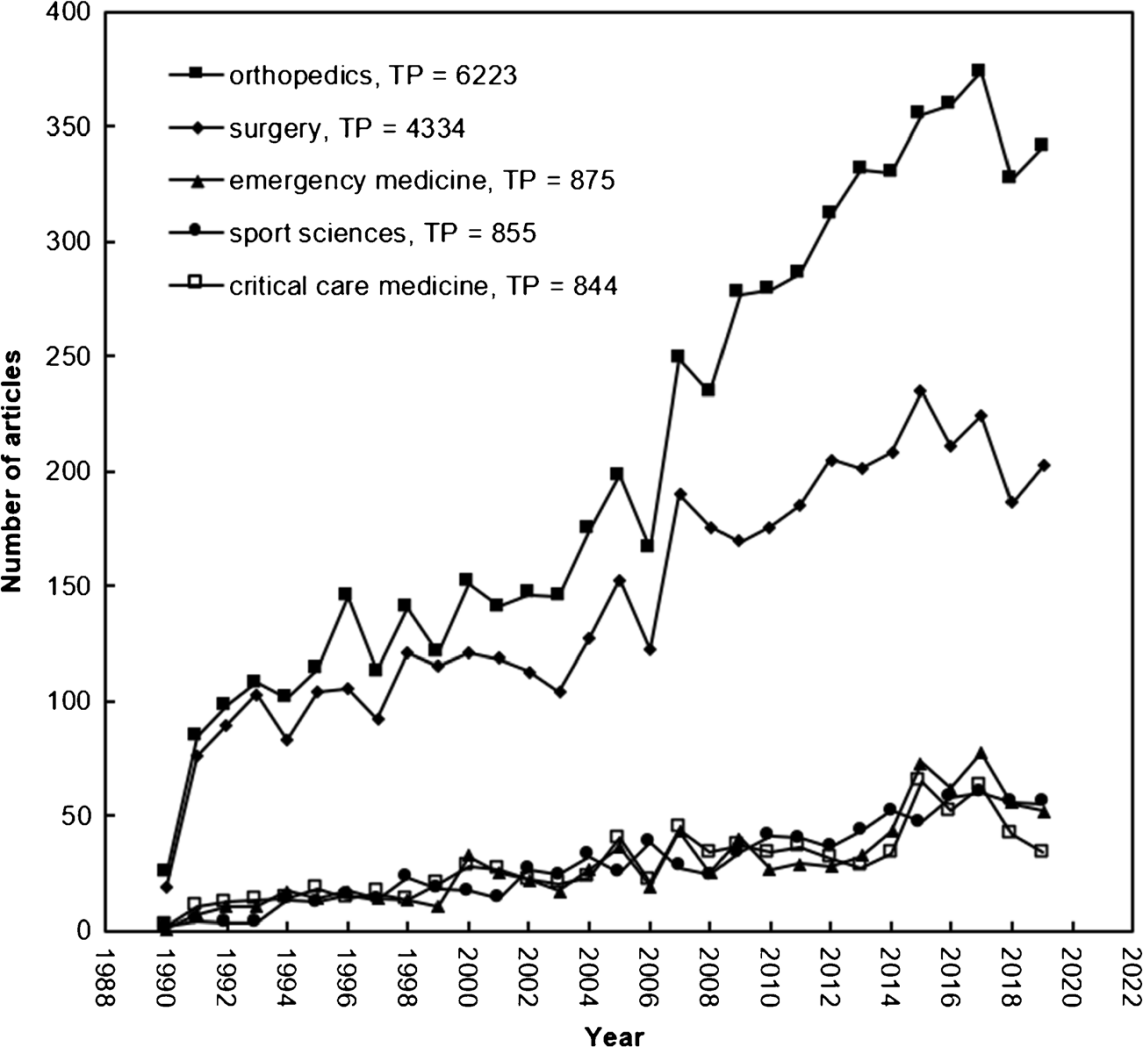
Comparison of the development nonunion articles trend of the top five Web of Science categories

Journal Citation Reports (JCR) indexed 9381 journals across 178 Web of Science categories in SCI-EXPANDED in 2019. A total of 8976 nonunion-related articles in SCI-EXPANDED were published in 790 journals which are classified among the 115 Web of Science categories in SCI-EXPANDED. Table 2 shows the top ten productive Web of Science categories with number of articles (TP), APP, CPP_2019_ and number of journals in a category. A total of 7407 articles (83% of 8975 articles with category information in SCI-EXPANDED) were published in the top two categories: orthopaedics (6223 articles; 69% of 8975 articles) and surgery (4334 articles; 48%). Comparing the top ten productive categories in healthcare sciences, articles published in category of clinical neurology had the highest CPP_2019_ of 34, while articles in general and internal medicine had lower CPP_2019_ of 6.1. Articles published in category of biomedical engineering had the highest APP of 6.5, while articles in paediatrics had lower APP of 4.2. The evolution of nonunion article trends in the top five productive Web of Science categories is shown in Fig. 3. Articles in categories of orthopaedics and surgery had similar trends from 1990 to 2008; however, orthopaedics shows a sharper increasing trend since 2009. The number of articles published, as well as trends in the remaining three categories, is similar.

**Table 2:** The top 10 productive nonunion Web of Science categories in SCI-EXPANDED

Web of Science category	*TP* (%)	No. *J*	*APP*	*CPP*_2019_
Orthopaedics	6223 (69)	82	4.4	23
Surgery	4334 (48)	210	4.4	25
Emergency medicine	875 (10)	31	4.4	17
Sport sciences	855 (10)	85	4.5	24
Critical care medicine	844 (9.4)	36	4.6	23
Clinical neurology	501 (5.6)	204	5.2	34
General and internal medicine	260 (2.9)	165	4.8	6.1
Paediatrics	227 (2.5)	128	4.2	15
Research and experimental medicine	209 (2.3)	138	6.0	14
Biomedical engineering	198 (2.2)	87	6.5	25

*TP*, number of publications; *APP*, number of authors per publication; *CPP*_2019_, number of citations (*TC*_2019_) per publication (*TP*); No. *J*, number of journals in a Web of Science category

The top ten most productive journals are listed in Table 3, including IF_2019_, APP, CPP_2019_ and Web of Science category. All the top ten journals were classified in the category of orthopaedics and six in surgery. The Injury-International Journal of the Care of the Injured published the most articles (657 articles; 7.3% of 8976 articles). Comparison of the articles published in the top ten journals showed that articles published in the Spine had the highest APP of 5.2, while the Journal of Hand Surgery-American Volume and the Clinical Orthopaedics and Related Research had an APP of 3.8, respectively. Articles published in the Journal of Bone and Joint Surgery-American Volume had the highest CPP_2019_ of 71, while articles published in International Orthopaedics had a CPP_2019_ of 14. According to IF_2019_, the top three articles have an IF_2019_ of more than 30 (Lancet, Nature Biotechnology, and BMJ-British Medical Journal).

**Table 3:** The top 10 productive journals with nonunion articles

Journal	*TP* (%)	*IF*_2019_	*APP*	*CPP*_2019_	Web of Science category
Injury-International Journal of the Care of the Injured	657 (7.3)	2.106	4.5	21	Critical care medicine Emergency medicine Orthopaedics Surgery
Clinical Orthopaedics and Related Research	470 (5.2)	4.329	3.8	38	Orthopaedics Surgery
Journal of Orthopaedic Trauma	466 (5.2)	1.897	4.6	29	Orthopaedics Sport sciences
Journal of Bone and Joint Surgery-American Volume	332 (3.7)	4.578	4.9	71	Orthopaedics Surgery
Journal of Hand Surgery-American Volume	290 (3.2)	2.124	3.8	25	Orthopaedics Surgery
Foot & Ankle International	264 (2.9)	2.292	4.1	22	Orthopaedics
Archives of Orthopaedic and Trauma Surgery	235 (2.6)	2.021	4.6	15	Orthopaedics Surgery
International Orthopaedics	233 (2.6)	2.854	4.8	14	Orthopaedics
Spine	184 (2.0)	2.646	5.2	49	Clinical neurology Orthopaedics
Journal of Bone and Joint Surgery-British Volume	182 (2.0)	IF_2014_=3.309	4.0	48	Orthopaedics Surgery

*TP*, number of publications; *IF*_2019_, journal impact factor in 2019; *APP*, number of authors per publication; *CPP*_2019_, number of citations (*TC*_2019_) per publication (*TP*)

### Publication performance of countries, institutions and authors

The 8976 articles were published by 26,079 authors among 101 different countries. Altogether, 7976 (89% of 8976 articles) were authored from a single country, with the articles being from 79 different countries. Internationally, authored collaborative articles were less common (993 articles, 11%) with authors from 85 different countries. The six publication indicators [10] were applied to compare the top ten productive countries (Table 4). Five European countries, three Asia countries and two American countries were ranked in the top ten of publications. The USA dominated the top in the six publication indicators with a TP of 3262 articles (36% of 8969 articles), an IP of 2726 articles (34% of 7976 single-country authored articles), a CP of 536 articles (54% of 993 internationally collaborative articles), an FP of 2996 articles (33% of 8969 first-author articles), an RP of 2939 articles (33% of 8832 corresponding-author articles) and an SP of 148 articles (39% of 378 single-author articles).

**Table 4:** Top 10 countries with most nonunion publications

Institute	*TP*	*TPR* (%)	*IPR* (%)	*CPR* (%)	*FPR* (%)	*RPR* (%)	*SPR* (%)
USA	3262	1 (36)	1 (34)	1 (54)	1 (33)	1 (33)	1 (39)
Germany	811	2 (9.0)	2 (7.5)	2 (22)	2 (7.8)	2 (8.0)	3 (6.1)
UK	701	3 (7.8)	4 (6.8)	3 (16)	4 (6.9)	4 (7.0)	3 (6.1)
China	667	4 (7.4)	3 (7.3)	6 (8.8)	3 (7.0)	3 (7.0)	11 (1.9)
France	447	5 (5.0)	5 (4.6)	8 (8.0)	5 (4.6)	5 (4.6)	6 (4.5)
Japan	389	6 (4.3)	6 (4.3)	12 (4.3)	6 (4.0)	6 (4.0)	15 (1.3)
Canada	331	7 (3.7)	10 (2.5)	4 (13)	8 (3.0)	8 (2.9)	7 (2.9)
South Korea	312	8 (3.5)	7 (3.4)	12 (4.3)	7 (3.3)	7 (3.3)	21 (0.79)
Italy	271	9 (3.0)	11 (2.4)	7 (8.1)	10 (2.5)	10 (2.5)	28 (0.26)
Switzerland	269	10 (3.0)	13 (1.7)	4 (13)	13 (2.0)	13 (2.0)	8 (2.4)

*TP*, total number of articles; *TPR* (%), the rank and the percentage of total articles in the total number of articles; *IPR* (%), the rank and the percentage of single-country articles in the total country independent articles; *CPR* (%), the rank and the percentage of internationally collaborative articles in the total internationally collaborative articles; *FPR* (%), the rank and the percentage of first-author articles in the total first-author articles; *RPR* (%), the rank and the percentage of the corresponding-author articles in the total corresponding-author articles; *SPR* (%), the rank and the percentage of the single-author articles in the total single-author articles; N/A, not available

In total, 4192 articles (47% of 8969 articles) were articles authored within single institution and 4777 (53%) were inter-institutionally collaborative articles. The six publication indicators [10] were applied to compare the top 11 productive institutions with TP of 70 or more (Table 5). Nine of the top 11 most productive institutions are in the USA and one in Canada and China, respectively. The Mayo Clinic in the USA took the leading position for the four publication indicators with a TP of 134 articles (1.5% of 8969 articles), an IP of 67 articles (1.6% of 4192 single institute articles), an FP of 92 articles (1.0% of 8969 first-author articles) and a RP of 93 articles (1.1% of 8,831 corresponding-author articles), while the University of Toronto in Canada ranked top with a CP of 82 articles (1.7% of 4777 inter-institutionally collaborative articles). The Chang Gung Memorial Hospital in Taiwan published the most single-author article with an SP of nine articles (2.4% of 378 single-author articles). The Hospital for Special Surgery in the USA, the New York University (NYU) in the USA, the Washington University in the USA and the Shanghai Jiao Tong University in China had no single-author articles.

**Table 5:** Top 11 institutions publishing nonunion-related articles with their author characteristics (*TP* ≥ 70)

Institute	*TP*	*TPR* (%)	*IPR* (%)	*CPR* (%)	*FPR* (%)	*RPR* (%)
Mayo Clinic, USA	134	1 (1.5)	1 (1.6)	4 (1.4)	1 (1.0)	1 (1.1)
Hospital for Special Surgery, USA	104	2 (1.2)	8 (0.62)	2 (1.6)	5 (0.61)	4 (0.60)
University of Toronto, Canada	103	3 (1.1)	14 (0.50)	1 (1.7)	9 (0.51)	13 (0.40)
Massachusetts General Hospital, USA	92	4 (1.0)	12 (0.55)	3 (1.4)	2 (0.65)	3 (0.65)
Harvard University, USA	89	5 (1.0)	5 (0.67)	6 (1.3)	10 (0.50)	10 (0.48)
New York University (NYU), USA	87	6 (1.0)	11 (0.57)	5 (1.3)	4 (0.64)	6 (0.54)
Washington University, USA	83	7 (0.93)	8 (0.62)	8 (1.2)	7 (0.54)	7 (0.53)
University of Washington, USA	80	8 (0.89)	13 (0.52)	7 (1.2)	18 (0.35)	16 (0.36)
Duke University, USA	79	9 (0.88)	6 (0.64)	10 (1.1)	11 (0.49)	8 (0.51)
Shanghai Jiao Tong University, China	70	10 (0.78)	3 (1.0)	24 (0.63)	6 (0.60)	4 (0.60)
University of California San Francisco, USA	70	10 (0.78)	18 (0.41)	9 (1.1)	16 (0.41)	16 (0.36)

*TP*, total number of articles; *TPR* (%), the rank and the percentage of total articles in the total number of articles; *IPR* (%), the rank and the percentage of institutional independent articles in the total institutional independent articles; *CPR* (%), the rank and the percentage of inter-institutionally collaborative articles in the total inter-institutionally collaborative articles; *FPR* (%), the rank and the percentage of first-author articles in the total first-author articles; *RPR* (%), the rank and the percentage of the corresponding-author articles in the total corresponding-author articles; *SPR* (%), the rank and the percentage of the single-author articles in the total single-author articles

In experimental science, the accepted convention is that the most important positions are first and last authors and one of these usually includes the corresponding author [6]. The first author generally is the person who contributed most to the work, including conducting research and writing of the manuscript [6]. The corresponding author is perceived as the author contributing significantly to the article independently of the author position [15]. The corresponding author supervised the planning and execution of the study and the writing of the paper [16]. Table 6 listed the top ten most productive authors. P.V. Giannoudis at the University of Leeds in the UK published the most nonunion articles followed by J.B. Jupiter at the Massachusetts General Hospital in the USA and C.C. Wu at the Chang Gung University in Taiwan. Publication performance of authors was further analysed with the Y-index which is related to the number of first-author publications (FP) and corresponding-author publications (RP). The Y-index combines two parameters (j, h), to evaluate the publication potential and contribution characteristics as a single index. This indicator has been used to compare authors in medical topics, such as occupational therapy [17], child sexual abuse [13] and breast reconstruction [6]. The Y-index is defined as [7]:

1 $$ j= FP+ RP $$

2 $$ h={\tan}^{-1}\left(\frac{RP}{FP}\right) $$

where *j* is the publication potential, a constant related to publication quantity, and *h* is publication characteristics which can describe the proportion of RP to FP. The greater the value of *j*, the greater the number of first- and corresponding-author articles by the author. Different values of *h* represent different proportions of corresponding-author articles from first-author articles.

- *h* = π/2, *j* is the number of corresponding-author articles.
- π/2 > *h* > 0.7854 indicates more corresponding-author articles.
- *h* = 0.7854 indicates the same number of first- and corresponding-author articles.
- 0.7854 > *h* > 0 indicates more first-author articles.
- *h* = 0, *j* is the number of first-author articles.

**Table 6:** Top ten most productive authors

Author	Rank (*TP*)	Rank (*FP*)	*FP CPP*_2019_	Rank (*RP*)	*RP CPP*_2019_	Rank (*SP*)	*SP CPP*_2019_	Rank (*j*)
P.V. Giannoudis	1 (50)	5 (12)	60	1 (35)	49	N/A	N/A	3 (46)
J.B. Jupiter	2 (48)	22 (7)	40	25 (10)	32	9 (2)	47	26 (15)
C.C. Wu	3 (42)	1 (36)	18	1 (35)	18	1 (15)	13	1 (70)
D. Ring	4 (41)	2 (23)	51	3 (30)	41	5 (3)	80	2 (50)
A.T. Bishop	5 (40)	1178 (1)	92	6 (14)	31	N/A	N/A	26 (15)
G. Schmidmaier	6 (39)	37 (6)	61	35 (8)	50	N/A	N/A	34 (14)
D.L. Helfet	7 (37)	180 (3)	62	33 (9)	27	N/A	N/A	71 (10)
E.H. Schemitsch	8 (36)	N/A	N/A	50 (7)	24	N/A	N/A	163 (7)
M. Bhandari	9 (34)	13 (9)	93	13 (12)	66	N/A	N/A	12 (19)
K.A. Egol	9 (34)	22 (7)	105	4 (25)	16	N/A	N/A	4 (32)

*TP*, total number of articles; *FP*, number of first-author articles; *RP*, number of corresponding-author articles; *SP*, number of single-author articles; *j*, constant of *Y*-index; *CPP*_2019_, citations (*TC*_2019_) per publication (*TP*); N/A, not available

Y-index was applied to evaluate the authors in 8551 (95%) of the 8976 nonunion articles with both of first-author and corresponding-author information in SCI-EXPANDED.

**Fig. 4:**
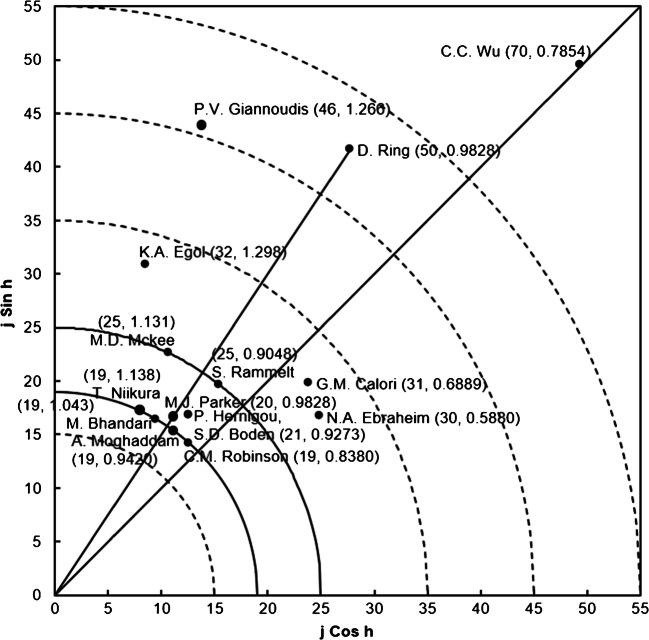
Distribution of the top 15 authors publishing nonunion articles with their *Y*-index values (*j* ≥ 19)

A total of 8551 articles were published by 25,219 authors in which 16,991 authors (67% of 25,219 authors) had no first- or corresponding-author articles with Y-index = (0, 0); 1596 (6.3%) authors published only corresponding-author articles with h = π/2; 446 (1.8%) authors published more corresponding-author articles with π/2 > *h* > 0.7854; 3613 (14%) authors published the same number of first- and corresponding-author articles with *h* = 0.7854; 242 (1.0%) authors published more first-author articles with 0.7854 > *h* > 0; and 2331 (9.2%) authors published only first-author articles with *h* = 0. Figure 4 shows distribution of the Y-index (*j*, *h*) of the top 15 authors with *j* ≥ 19. Each dot represents one value of the Y-index that could be one author or many authors, for example, P. Hernigou and S.D. Boden who share the same Y-index = (21, 0.9273). C.C. Wu from the Chang Gung University in Taiwan had the highest publication potential with a *j* of 70 and Y-index = (70, 0.7854). Wu published 42 nonunion articles including 36 first-author articles and 35 corresponding-author articles (Table 6). Wu published not only the most first-author articles and the corresponding-author articles but also the most single-single articles. However, Wu’s nonunion articles had lower CPP_2019_, followed by D. Ring from the Massachusetts General Hospital of Harvard University in the USA as the corresponding affiliation with a *j* of 50 and Y-index = (50, 0.9828) and P.V. Giannoudis from the University of Leeds in the UK with a *j* of 46 and Y-index = (46, 1.266). Comparing the top three authors had higher publication potential in nonunion articles: P.V. Giannoudis had higher CPP_2019_ of his first-author articles and corresponding-author articles than Wu and Ring, while Ring had higher CPP_2019_ for his single-author articles (Table 6). M.D. McKee with Y-index = (25, 1.131) and S. Rammelt with Y-index = (25, 0.9048) both had a *j* of 25. McKee and Rammelt are located on the same curve (*j* = 25) in Fig. 4, indicating that they had the same publication potential with the same value of ***j*** but different publication characteristics [18]. Similarly, T. Niikura (19, 1.138), M. Bhandari (19, 1.043), A. Moghaddam (19, 0.9420) and C.M. Robinson (19, 0.8380) are also located on the same curve (*j* = 19). Niikura published higher ratio of the number of corresponding-author articles to the number of first-author articles with an *h* of 1.138, followed by Bhandari with an *h* of 1.043, Moghaddam with an *h* of 0.9420 and Robinson with an *h* of 0.8380. These authors had the same publication potential with a *j* of 19 but the publication characteristics were also different. D. Ring with Y-index = (50, 0.9828) and M.J. Parker with Y-index = (20, 0.9828) are located on the line (*h* = 0.9828), indicating that they had the same publication characteristics but different publication potentials [19]. Ring had the greater publication potential with a *j* of 50 than Parker with a *j* of 20. It has been pointed out that a bias in analysis of authorship might occur when different authors had the same name, or one author used different names (e.g. maiden names) in their articles [20].

### Top ten most frequently cited nonunion-related articles

The total number of citations of a document in the Web of Science Core Collection is updated from time to time. Ho’s group proposed citation indicators TC_year_ [21] and C_year_ [7]. The advantage of using TC_year_ and C_year_ is that they are immutable and ensure repeatability compared with the citation index of the Web of Science Core Collection [22]. Citation frequency is considered to reflect the impact of scientific publications, although this does not necessarily correlate with the paper quality [23]. The best articles can be classified as articles that most researchers can read and cite in peer-reviewed journals [24]. The 8976 nonunion articles ranked differently if sorted by TC_2019_ than sorted by C_2019_. A total of 3521 articles (39% of 8976 articles) had no citation in the most recent year (C_2019_ = 0) and 987 (11%) articles had no citation from their publication year to the end of 2019 (TC_2019_ = 0). Furthermore, among the top 100 C_2019_ articles, 34% of the articles were among the top 100 TC_2019_ articles.

**Fig. 5:**
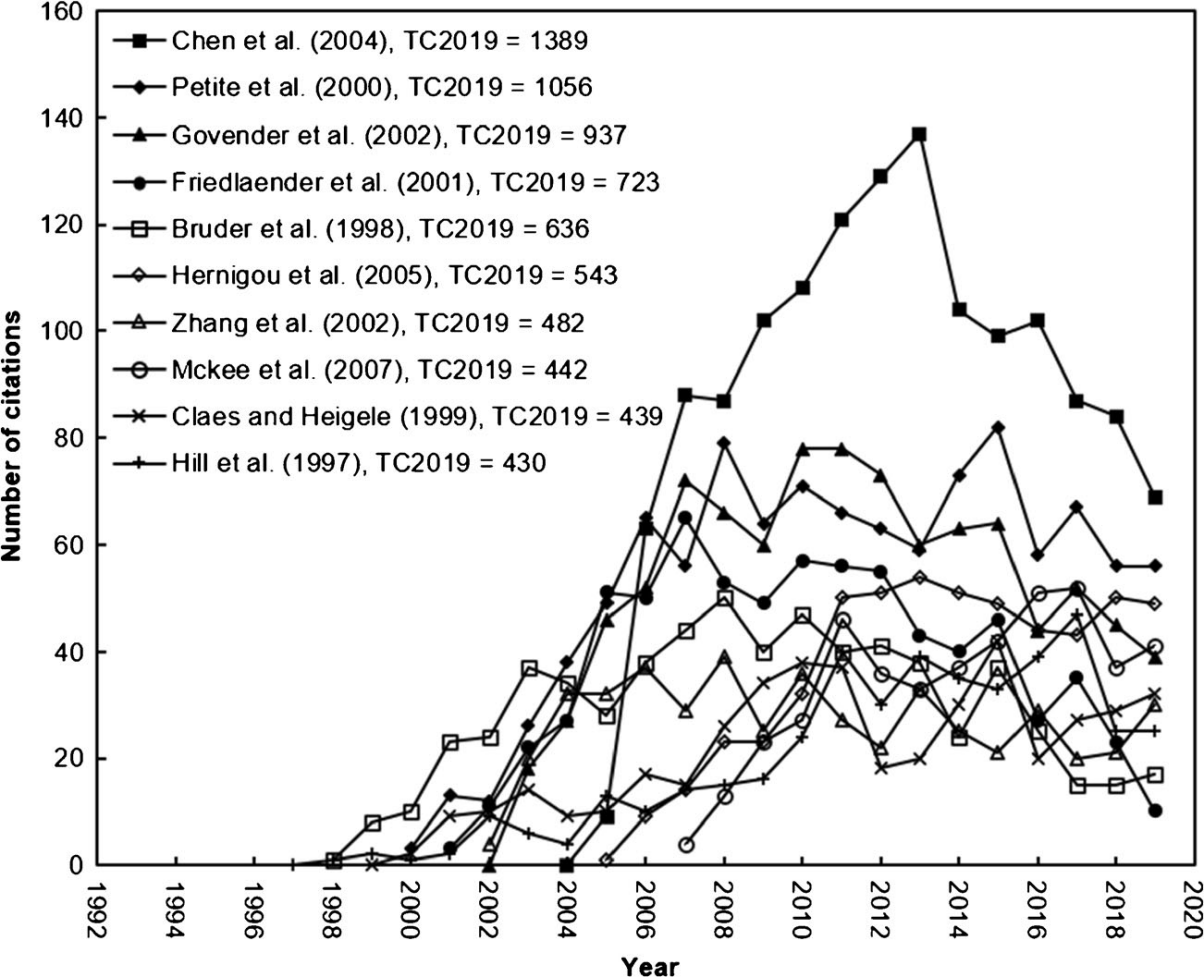
The citation history of the top ten most frequently cited nonunion articles

The 8976 nonunion articles were searched out with search keywords on their ‘front page’: title, abstract and author keywords from SCI-EXPANDED in the last three decades. Table 7 lists the ten most frequently cited articles in nonunion research with two citation indicators [7]. Five of the ten articles were published in the Journal of Bone and Joint Surgery-American Volume (IF_2019_ = 4.578). Nine of the ten articles were single-country articles from the USA with five articles, France with two and one by Canada and Germany, respectively. The only internationally collaborative article was published by 59 authors from South Africa, the USA, Israel, Finland, the UK, Germany, France, Australia, Canada, Belgium, Netherlands and Norway. The citation history of the top ten most frequently cited articles is presented in Fig. 5. The article by Chen et al. [25] had a sharply increased citation after its publication for ten years in nonunion studies and then decreased. It ranked top on annual citation from 2007 to 2019. An article’s impact might not be always high [22]. A highly cited article published by Friedlaender et al. (2001) had a TC_2019_ of 723 ranked fourth but had a low impact in 2019 with a C_2019_ of ten (ranked 217th) indicating that with time, the significance of the article diminished. Although some recently published articles in the past few years have great potential, their TC_2019_ is not high. The top three cited articles in 2019 by Campana et al. (2014), Xavier et al. (2015) and Goodman et al. (2013) had a C_2019_ of 117 (ranked 1st), 77 (ranked 2nd) and 73 (ranked 3rd) but had a low TC_2019_ of 262 (ranked 24th), 220 (ranked 41st) and 387 (ranked 14th).

**Table 7:** The ten most frequently cited articles in nonunion-related articles

Rank (*TC*_2019_)	Rank (*C*_2019_)	Title	Country	Reference
1 (1389)	4 (69)	Bone morphogenetic proteins	USA	Chen et al. [25]
2 (1056)	5 (56)	Tissue-engineered bone regeneration	France	Petite et al. [26]
3 (937)	10 (39)	Recombinant human bone morphogenetic protein-2 for treatment of open tibial fractures: A prospective, controlled, randomized study of four hundred and fifty patients	South Africa, USA, Israel, Finland, UK, Germany, France, Australia, Canada, Belgium, Netherlands, Norway	Govender et al. [27]
4 (723)	217 (10)	Osteogenic protein-1 (bone morphogenetic protein-7) in the treatment of tibial nonunions: A prospective, randomized clinical trial comparing rhOP-1 with fresh bone autograft	USA	Friedlaender et al. [28]
5 (636)	55 (17)	The effect of implants loaded with autologous mesenchymal stem cells on the healing of canine segmental bone defects	USA	Bruder et al. [29]
6 (543)	6 (49)	Percutaneous autologous bone-marrow grafting for nonunions: Influence of the number and concentration of progenitor cells	France	Hernigou et al. [30]
7 (482)	17 (30)	Cyclooxygenase-2 regulates mesenchymal cell differentiation into the osteoblast lineage and is critically involved in bone repair	USA	Zhang et al. [31]
8 (442)	9 (41)	Nonoperative treatment compared with plate fixation of displaced midshaft clavicular fractures: A multicenter, randomized clinical trial	Canada	Society COT et al. [32]
9 (439)	16 (32)	Magnitudes of local stress and strain along bony surfaces predict the course and type of fracture healing	Germany	Claes and Heigele [33]
10 (430)	21 (25)	Closed treatment of displaced middle-third fractures of the clavicle gives poor results	USA	Hill et al. [34]

*TC*_2019_, the total number of citations from Web of Science Core Collection since publication year to the end of 2019; *C*_2019_, the number of citations of an article in 2019 only

Five articles were ranked on both the top 10 TC_2019_ as the most frequently cited articles and C_2019_ as the most impact in the most recent year articles. These five highly cited and the most impact articles in 2019 articles were summarised as follows:

1. Bone morphogenetic proteins [25]

C_2019_ of 69 (ranked 4th) and TC_2019_ of 1389 (ranked 1st)

In this article, Chen et al. [25] was one of the first to provide a state-of-the-art review of the advancements on basic science and clinical applications of bone morphogenetic protein (BMP) research. BMPs are extremely important in embryonic development as well as in postnatal cell functions and are involved in the heart, nervous system and cartilage development, as well as postnatal bone formation with strong bone induction properties. Therefore, at that early stage, numerous potential therapeutic applications of BMPs were outlined including bone defects and fracture nonunion. In the years following this publication, BMPs were widely studied and successfully applied in the treatment of nonunions with BMP-2 and BMP-7 currently approved for use by the US Food and Drug Administration [35] and are considered critical components in the therapeutic armamentarium for nonunions [36].

2. Tissue-engineered bone regeneration [26]

C_2019_ of 56 (ranked 5th) and TC_2019_ of 1056 (ranked 2nd)

In this pioneer basic science paper, the authors manufactured a combined construct of coral scaffold and marrow stromal cells (MSCs) to fill bony defects in sheep. Contrary to the failure of scaffolds used in the past, the idea was to use the naturally occurring coral exoskeleton which has adequate mechanical properties and increased porosity similar to cancellous bone, in order to better deliver the MSCs. The authors established ‘critical’-size bone defects of increasing width in sheep metatarsals and treated the defects with either coral exoskeleton alone, or in combination with fresh bone marrow or MSCs. They succeeded in bridging the defects after 16 weeks in the third group of combined coral exoskeleton and MSCs and this was the first study to effectively demonstrate replacement of a critical defect with mature bone that would have evolved into a nonunion otherwise.

3. Percutaneous autologous bone-marrow grafting for nonunions: Influence of the number and concentration of progenitor cells [30]

C_2019_ of 49 (ranked 6th) and TC_2019_ of 534 (ranked 6th)

This study was the first one to provide guidelines and revive interest on the harvesting of bone marrow aspirate concentrate (BMAC) from the iliac crest and its subsequent injection in atrophic tibia nonunion sites [30]. Following bone marrow aspiration from bilateral iliac crests and its concentration via a cell separator, 20 cc of BMAC were injected into the nonunion site of 60 patients. Fifty-three patients went into union, whereas seven developed nonunion. Although the study confirmed the efficacy of the procedure from previous reports [37], it was the first to show that the number of progenitor cells as well as the need for increased concentration is critical in order to obtain union. From that point onward, BMAC in the treatment of nonunions has been widely used and the area is still under constant evolution [38].

4. Non-operative treatment compared with plate fixation of displaced midshaft clavicular fractures: a multicenter, randomized clinical trial [39]

C_2019_ of 41 (ranked 9th) and TC_2019_ of 442 (ranked 8th)

Prior to this study, even the most displaced midshaft clavicle fractures, were traditionally receiving non-operative treatment based on earlier reports [40] that had inherent biases. However, some subsequent studies showed increased level of complications such as nonunion and malunion as well as lower rates of patient satisfaction [41]. This prospective randomised clinical trial coming from eight Canadian centres and published by the Canadian Orthopaedic Association looked at 132 patients divided into two groups of similar characteristics and who received either plate fixation or non-operative treatment with a sling [39]. The authors looked at time to radiographic union, constant shoulder scores, DASH scores, complications and patient satisfaction at one year. Plate fixation showed better outcomes as far as function (scores) and malunion/nonunion complications, as well as patient satisfaction. In the plate fixation group, three out of 67 patients developed a wound infection but were successfully addressed with local wound care and antibiotics, and later plate removal with formal irrigation/debridement after union. The authors concluded that primary plate fixation of those injuries is justified. Although this study significantly helped revive the debate of operative versus non-operative management of displaced midshaft clavicle fractures, it is still unclear what the best option is, and patients must be adequately informed of the pros and cons of each treatment, as outlined in a recent systematic review also from Canada [42].

5. Recombinant human bone morphogenetic protein 2 for treatment of open tibial fractures: a prospective, controlled, randomized study of four hundred and fifty patients [27]

C_2019_ of 39 (ranked 10th) and TC_2019_ of 937 (ranked 3rd)

This multi-author randomised control trial by the BMP-2 Evaluation in Surgery for Tibial Trauma (BESTT) study group evaluated the safety and efficacy of the use of recombinant BMP-2 (rhBMP-2) in the treatment of open tibial shaft fractures compared to the standard of care [27]. Τhe authors looked at 121 patients with a 12-month follow-up receiving either intramedullary nail alone with routine wound care or intramedullary nailing with addition of an implant consisting of an absorbable collagen sponge containing rhBMP-2 of 6 or 12 mg over the fracture site. The study found that the patients who received the 12-mg dose rhBMP-2 implant had lower rates of hardware failure and infections and demonstrated faster wound healing and required fewer number of secondary interventions than the other two groups. Since then, there have been several clinical studies showing that rhBMP-2 (as well as rhBMP-7) enhances bone defect regeneration in adults and as stated earlier they have been approved by the US Food and Drug Administration for specific clinical indications [35].

### Research focuses and their development trends

**Fig. 6:**
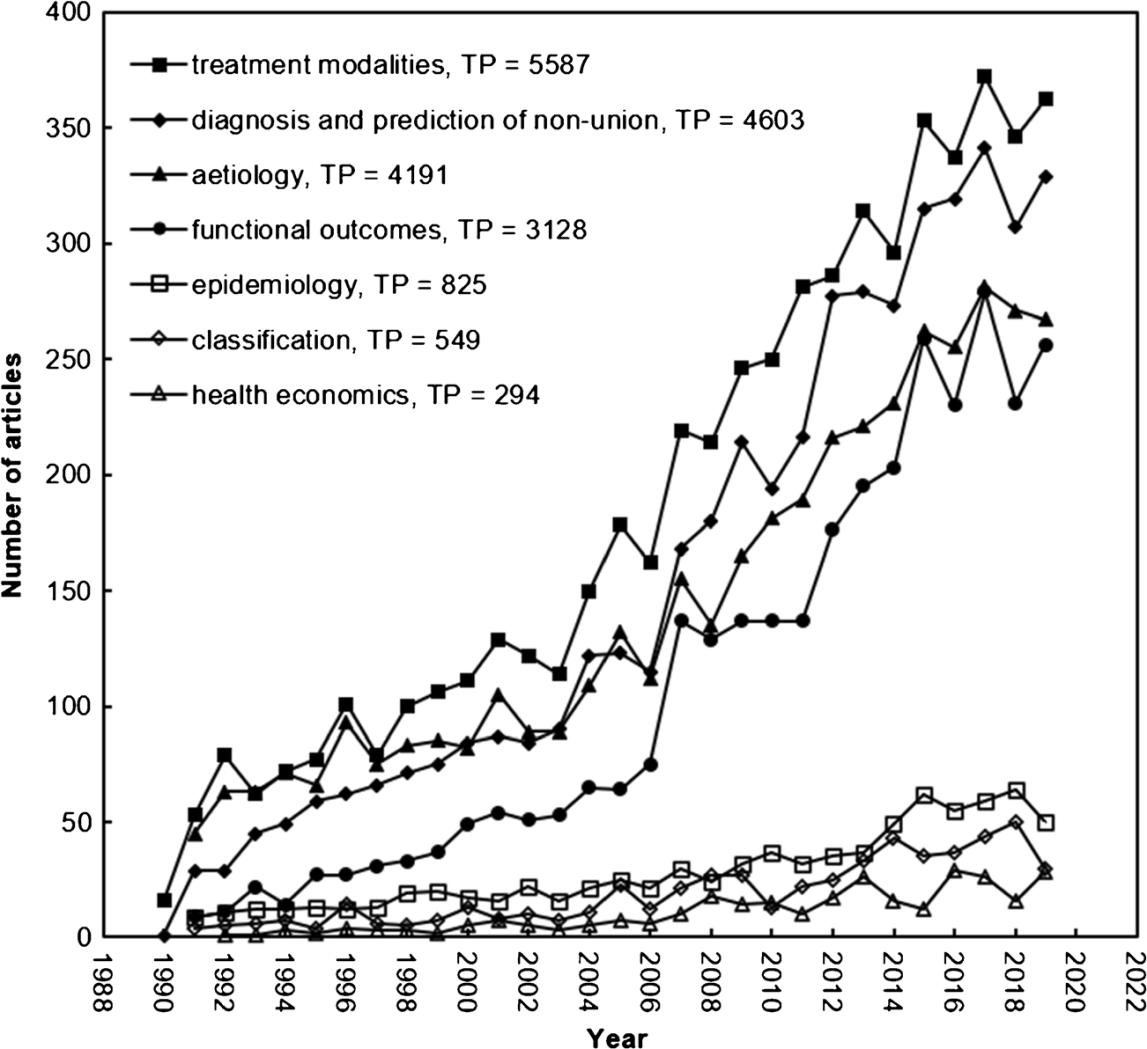
Development of the seven main foci

Ho’s group proposed distribution of words in article titles, abstracts, author keywords and KeyWords Plus in different periods as information to evaluate main research focuses and find their development trends in research topics [43]. The results of keyword analyses provide information about the main and possible research foci as each word cluster comprised several supporting words. Thus, the development of the seven possible main research foci in nonunion-related research is presented. Figure 6 shows the development of the seven topics.

- *Topic 1: Epidemiology*
  Supporting words—epidemiology, incidence, incidences, prevalence and prevalences
- *Topic 2: Aetiology (or etiology, or cause)*
  Supporting words—aetiology, cause, causes, caused, factor, factors, heal, heals, infection, infections, infectious, infected and etiology
- *Topic 3: Classification*
  Supporting words—classification and classifications
- *Topic 4: Diagnosis and prediction of nonunion*
  Supporting words—predict, predictable, predictably, predicting, prediction, predictive, predictor, predictors, predicts, score, scoring, scores, diagnose, diagnoses, diagnosing, diagnosis, diagnostic, diagnostics, radiographic, radiographs, radiography, radiologic, radiological, radiotherapy, radioulnar, biomarkers, mark, markedly, marker and markers
- *Topic 5: Treatment modalities*
  Supporting words—treat, treating, treatment, treatments, management, manage and managing
- *Topic 6: Functional outcomes*
  Supporting words—outcome and outcomes
- *Topic 7: Health economics*
  Supporting words—cost, costs, economic, economical, economically, economics, burden and burdens

These seven topics identified support the efforts of clinicians over the years to understand better the characteristics, aetiology, diagnosis, prediction and treatment options that can be applied successfully to treat this difficult complication. Moreover, the burden of this condition, on the physiological and mental state of patients, has received a lot of attention. Finally, the cost-related implications of treatment have also been explored. All of the above themes represent the spectrum of research activities that have taken place up to now. It is envisaged that in the future, the same themes of research on nonunion will continue to dominate the activities of the scientific and clinical community.

## Limitations

Bibliometrics represents a quantitative method of publications, allowing researchers and scientists to assess numerous, unlimited peer-reviewed publications in a specific field of science. It is based on the citation number of existing publications. Interestingly, it should be emphasised that there is no correlation between the frequency of citations and the quality of the research published. In addition, due the inherent delays between publishing an articles and breakthroughs and innovations in treatment modalities, high-cited papers identified in bibliometric analysis may not represent the latest technological advances applied in the clinical setting. While bibliometric analysis can identify trends of research, specific institutions with focused research programmes and outputs in a topic of interest, it cannot provide evidence in terms of guidelines of treatment. Strengths of bibliometrics include mapping of the literature, identifying key opinion leaders and development of networking for collaborative research.

## Conclusions

A bibliometric analysis of the literature was carried out on fracture nonunion which is the most common fracture fixation complication in the field of orthopaedic trauma. Overall, over a 30-year period, 10,064 nonunion-related publications were published. Such information was generated as number of citations, publications outputs, journals published the articles, institutions and countries involved and focus of topics related to fracture nonunion (epidemiology, classification, diagnosis, prediction, treatment modalities, functional outcomes and cost of treatment). It is envisaged that the information contained in this study will assist clinicians and scientist to shape their research focus in the future and by having identified prominent researchers and institutions and help stimulate international team collaborative efforts.
